# A Review of Vector-Borne Rice Viruses

**DOI:** 10.3390/v14102258

**Published:** 2022-10-14

**Authors:** Pengyue Wang, Jianjian Liu, Yajing Lyu, Ziting Huang, Xiaoli Zhang, Bingjian Sun, Pengbai Li, Xinxin Jing, Honglian Li, Chao Zhang

**Affiliations:** 1 Department of Plant Pathology, College of Plant Protection, Henan Agricultural University, Zhengzhou 450002, China; 2 Hubei Engineering Research Center for Pest Forewarning and Management, College of Agronomy, Yangtze University, Jingzhou 434025, China; 3 Co-Construction State Key Laboratory of Wheat and Maize Crop Science, College of Agronomy, Henan Agricultural University, Zhengzhou 450046, China

**Keywords:** rice viral diseases, disease symptoms, RNA silencing, plant hormones, antiviral defense, disease prevention and control

## Abstract

Rice (*Oryza sativa* L.) is one of the major staple foods for global consumption. A major roadblock to global rice production is persistent loss of crops caused by plant diseases, including rice blast, sheath blight, bacterial blight, and particularly various vector-borne rice viral diseases. Since the late 19th century, 19 species of rice viruses have been recorded in rice-producing areas worldwide and cause varying degrees of damage on the rice production. Among them, southern rice black-streaked dwarf virus (SRBSDV) and rice black-streaked dwarf virus (RBSDV) in Asia, rice yellow mottle virus (RYMV) in Africa, and rice stripe necrosis virus (RSNV) in America currently pose serious threats to rice yields. This review systematizes the emergence and damage of rice viral diseases, the symptomatology and transmission biology of rice viruses, the arm races between viruses and rice plants as well as their insect vectors, and the strategies for the prevention and control of rice viral diseases.

## 1. Introduction

Rice (*Oryza sativa* L.) is a vital food crop for the world’s human populations [1,2,3]. Since 1961, the start year of the Green Revolution, rice production has had a great improvement with the attempts of progressive breeding works and changes in cropping practices involving increased use of fertilizers, irrigation, machinery, and pesticides. Rice consumption has grown dramatically in many regions of the world in recent years, and the FAO speculates that by 2035, a 26% increase in rice production must be achieved to feed the growing population [4,5]. However, disease occurrence in rice plants poses a great threat to rice productivity, quality, and sustainability. Plant pathogens, including viruses, bacteria, fungi, and nematodes, together with diverse insect pathogens such as planthoppers, leafhoppers and chrysomelid beetles, caused about total 30% yield losses in staple food crops worldwide [6,7]. Sometimes, complete regional crop failures directly cause the challenge of plant disease-induced famine [8]. Notably, insect pathogens such as planthoppers and leafhoppers pose dual damage to rice yield because of their direct damage to rice growth and development in addition to their high efficiency in rice viral transmission, by which the food crisis in local or extensive rice-growing areas always occurs.

This review describes the emergence and impact of vector-borne viruses of rice in tropical and sub-tropical areas and discusses some of the factors influencing the apparent increase in prevalence of rice viral diseases. It systematically summarizes the symptomatology and transmission biology of rice viruses and the major achievements of the aspects on antiviral defense and virus counter-defense during the last two decades. It also outlines the measures being applied for disease prevention and control and prospects the rice viral disease management in the foreseeable future.

## 2. The Emergence and Damage of Various Rice Viruses Worldwide

**Figure 1:**
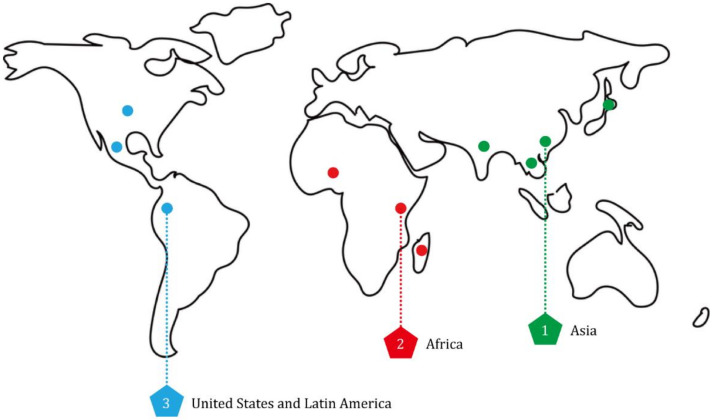
Global distribution of rice viral diseases. Three rice viral disease endemic areas are illustrated by circles with different colors. Green circles represent the main areas of rice viral diseases in Asia. Red circles represent the main areas of rice viral diseases in Africa. Blue circles represent the main areas of rice viral diseases in the United States and Latin America.

During the last half century, the improved productivity of rice has greatly alleviated food crisis caused by population growth, environmental stresses, diseases and pests [9,10,11]. But, with the development of rice breeding process, diverse vector-borne viral pathogens frequently emerged in many rice-growing areas and threatened high yield and quality of rice. There are three primary endemic regions of rice viral diseases: Asia, Africa, and America (Figure 1). Overall, most of these viruses are distributed in Asia, and only two have recurrently been reported in Africa and America, i.e., rice yellow mottle virus (RYMV) in Africa and rice hoja blanca virus (RHBV) in America (Table 1).

### 2.1. Rice Viral Diseases in Asia

The rice region of Asia has the largest variety of viral diseases and the highest frequency of occurrence. Until now, 15 viruses have been isolated from rice plants in Asia.

Records indicate that, before the Green Revolution, three rice viral diseases caused by rice dwarf virus (RDV), rice stripe virus (RSV), and rice black-streaked dwarf virus (RBSDV) were noticed and described sequentially in Japan. RDV was the first virus documented in rice plants in 1883 [10,11]. It causes rice dwarf disease sporadically. RDV-infected rice plants exhibit severe stunted growth, shorter and fewer roots, and poor grains [35,36], thus leading to dramatic reduction of rice yields. High local incidence of RDV was recorded in southern and central Japan during the period 1889–1979 and Zhejiang, China from 1969–1973 [11,37,38]. The high incidence of RDV was attributable to several reasons: the application of excess nitrogen fertilizer increased the vector population and the susceptibility of rice plants to RDV, the increase in areas planted for early rice raised vector density and the proportion of viruliferous vectors, the increase of rice fields left fallow during the winter season favored the overwintering of vector on weeds, and the developed resistance to pesticide of vector populations. RSV was initially discovered in Japan in 1897 [12]. In 1963, viral disease caused by RSV outbroke in the Jiangsu–Zhejiang–Shanghai (JZS) district of China [12], where rice production had severely suffered from RSV for a long period. Particularly during 2002–2004, RSV was circulated widely and severely in Yangtze River region and generally resulted in a 30–40% yield loss in eastern China [39,40,41,42]. This epidemic was largely due to the increased winter wheat production, which supports a large vector population in winter. In 1952, Japanese scientists determined RBSDV to be the agent for an emerging dwarf-associated rice disease [13]. RBSDV can naturally infect many Poaceae crops, including the three major stable crops: rice, maize, and wheat. RBSDV caused several outbreaks in China (1963; 1965–1967), Japan (1957–1961; 1965–1967), and Korea (1975–1976) [11]. It decimated almost 100% of the rice fields in some areas of Eastern China in 1997–1998. The high RBSDV incidence was mostly attributed to the promotion of early rice or increased wheat cultivation.

In 1963, rice grassy stunt virus (RGSV)-causing grassy stunt disease was first observed in the Philippines [14]. In the beginning, this disease occurred locally, but more than half of the rice plants were infected by RGSV from 1964–1965 in the Philippines. To date, it has been reported in many other rice-growing areas of Asia, including Indonesia, China, and Japan. It has been estimated that the combined effects of virus and vector caused losses exceeding 3.3 million tons of rice in Indonesia between 1974 and 1977 [43]. In Vietnam in 2009, more than 485,000 ha of paddy field were severely affected by infection with RGSV or co-infection of RGSV and rice ragged stunt virus (RRSV), resulting in the loss of 828,000 tons of rice valued at $1.2 × 10^8^ and directly affecting millions of rice farmers [44]. In 1963, rice tungro disease (RTD) in rice plants that simultaneously infected by rice tungro bacilliform virus (RTBV), a double-stranded DNA virus [15], and rice tungro spherical virus (RTSV), a single-stranded RNA virus [16] appeared in the countries of Southeast Asia, including China. RTD caused annual losses of more than $1.5 × 10^9^ in the 1980s [45]. In 1965, rice yellow stunt virus (RYSV), a member of the genus *Alphanucleo-rhabdovirus* in the family *Rhabdoviridae*, was first described in Taiwan and Guangdong provinces of China [18,19]. Major outbreaks of RYSV occurred in southern and central China, including Taiwan (1960–1962; 1973–1975; 1977–1980), Guangdong (1964–1966; 1979), Fujian (1966; 1969; 1973), and Zhejiang (1970–1972) provinces. Areas affected by RYSV in Japan and Thailand were localized with low incidence. Rice bunchy stunt virus (RBSV) caused disease in Fujian, Guangdong, Guangxi, Hubei, Hunan, Jiangxi, Guizhou, and Yunnan provinces, China, in the 1970s [20]. Average RBSV incidence reached the highest point in 1979, but it has disappeared lately in the field. Ragged stunt disease caused by RRSV was first reported at the International Rice Research Institute (IRRI) and elsewhere in the Philippines in 1977 [21]. It has since been discovered in many other Asian countries, including Thailand, China, and Vietnam, and has led to serious disease outbreaks in the rice-growing areas of these countries. In 1979, rice gall dwarf virus (RGDV), a member of the genus *Phytoreovirus* in the family *Sedoreoviridae*, was found in Thailand [22]. As of late, RGDV broadly infects rice plants in Southern China, including Fujian, Guangdong, Guangxi, and Hainan provinces. Rice necrosis mosaic virus (RNMV), a classic fungus-transmitted virus belonging to the genus *Bymovirus* in the family *Potyviridae*, was first reported in Japan and later in India [23,24]. It also infects some commercially important crops such as *Ludwigia perennis* and *Corchorus olitorius*, leading to an increase in both shoot growth and leaf size, along with characteristic chlorotic lesions on leaves [46,47,48].

From then to the year 2000, no novel virus was discovered in Asian rice-planting regions. However, the present rice viruses RBSDV, RSV, RGSV, and RRSV frequently caused outbreaks of viral disease in distinct rice-growing areas.

In 2001, a novel rice viral disease emerged in East and Southeast Asia [25,26]. It has been determined that southern rice black-streaked dwarf virus (SRBSDV), a new *Fijivirus* species in the family *Spinareoviridae*, is the agent of this disease. By 2009, rice fields in 19 provinces in northern Vietnam and 9 provinces in southern China had suffered from this disease. In 2010, more than 60,000 ha of paddy fields in 29 provinces of Vietnam and more than 1,300,000 ha in 13 provinces of China were infected, causing crop failure in many regions [49,50]. As of late, SRBSDV has become a dominant virus species that causes severe yield losses in many south and southeast Asian countries. In 2015, rice stripe mosaic disease (RSMD) was first discovered in the southwestern rice region of Guangdong province in China. The disease is caused by rice stripe mosaic virus (RSMV), a new cytorhabdovirus in the *Rhabdoviridae* family [27,28]. In the beginning (2015–2016), RSMD only occurred in southwestern Guangdong province in China; particularly in Luoding, where approximately 70% of the rice plants were affected by RSMD. From 2016 to 2018, this disease spread to Guangxi and Hainan provinces. From 2019 to now, RSMV-infected rice plants have also occasionally been observed in parts of Jiangxi, Hunan, and Yunnan provinces. These investigation data indicate that RSMD is gradually spreading throughout southern China [51]. In 2017, using next-generation sequencing technologies, a novel RNA virus, tentatively named rice virus A (RVA) was discovered from the collected rice samples with virus-like symptoms in the fields around South Korea [29]. In the past two years, a new picornavirus and a bunya-like virus were identified from rice plants exhibiting dwarfing or curling symptoms by high-throughput RNA-seq, tentatively named rice curl dwarf-associated virus (RCDaV) and rice dwarf-associated bunya-like virus (RDaBV), respectively [30,31]. These three tentatively named viruses are discovered from rice samples with virus-like symptoms in the fields. Whether these three viruses have potential for outbreaks in the rice-growing areas still warrants further investigation.

### 2.2. Rice Viral Diseases in Africa

Rice-growing countries in Africa form another center for viral disease epidemics. In 1966, yellow mottle disease was first reported in rice plants growing around Lake Victoria, Kenya. This disease is caused by RYMV, a sobemovirus transmitted by species of Chrysomelid beetles including *Chaetocnema pulla* [32]. The disease first appearing in Kenya was partially due to a new irrigation project, by which the rice cultivation dramatically increased with the benefit of availability of water for sequential plantings throughout the year. Later, rice yellow mottle disease spread to many other African countries where cropping practices were similar with Kenya, including Côte d’Ivoire, Liberia, Republic of Niger, Nigeria, Sierra Leone, and Tanzania. Since the early 1990s, RYMV had been present everywhere in sub-Saharan Africa and in Madagascar where rice was grown. It affected all types of rice cultivation, including lowland, upland, rain-fed, floating, and mangrove rice. The epidemic of rice yellow mottle disease in Africa is due to a buildup of infection associated with the introduction of irrigation and year-round cultivation [52]. For instance, some of the worst epidemics of yellow mottle disease have occurred in the Republic of Niger where the irrigated area increased from 571 ha in 1974 to 4,803 ha in 1984 and 8,500 ha in 1986 [53,54]. Aside from the cropping practices, the introduced lowland rice varieties from Asia also led to the serious outbreaks of rice yellow mottle disease. Because many upland accessions of the African rice *Oryza glaberrima* and local upland types of *O. sativa* are very tolerant to RYMV, Fomba considered that RYMV is an indigenous one to which local rice plants have adapted, whereas the apparent equilibrium established between host and virus seems to have been disrupted by the introduction of exotic varieties and an intensification of cropping practices [55].

RSNV, a soil-borne benyvirus transmitted by the fungus *Polymyxa graminis*, is an important causal agent that causes severe viral disease in rice-growing areas in both Africa and America. RSNV-causing disease was first described in 1977 as a new virus infecting rice in Côte d’Ivoire [33] and was subsequently observed in Liberia, Nigeria, and Sierra Leone [56]. RSNV was initially identified in the Ivory Coast in 1983 and the incidence of disease ranged locally from 37% to 80% in these African countries [34]. Since then, this virus was nearly undetected in the fields in Africa. However, it has been episodically reported in several South and Central American countries, including Colombia, Brazil, Ecuador, Panama, and Argentina [34,56,57,58,59,60], causing severe epidemics and leading to up to 40% yield losses [60]. Notably, characteristic symptoms of crinkling yellow color and deformation of rice leaves were recently observed in West African countries, including Burkina Faso [61], Benin [62], Mali [63], and Sierra Leone [64], suggesting that RSNV is re-emerging in Africa.

### 2.3. Rice Virus Diseases in America

In America, hoja blanca disease is a dominant viral disease in rice-growing regions. Rice hoja blanca virus (RHBV), a tenuivirus in the family *Phenviviridae*, is the causal agent for this disease. It was first noticed and described in Colombia in 1935 [10,11]. Since then, it has been reported in many other South and Central American countries and occasionally in the southern United States [65]. Until 1956, it seemed to have been sporadic and of limited importance, however, serious losses were subsequently reported in Cuba and Venezuela [66], of which total crop losses were estimated to have been 25% in Cuba and 50% in Venezuela. Severe problems were also encountered at the time in Colombia, Costa Rica, and Panama. Sometimes, RHBV can cause up to 100% yield losses in Latin American rice fields. The worst epidemics of hoja blanca disease were associated with three primary reasons: (1) the introduction and adoption of Bluebonnet 50 and other improved long-grained varieties [67]; (2) changes in cropping practices and the trend from one to two crops per year [68]; (3) the greatly increased populations of vectors.

## 3. Symptomatology

Susceptible rice plants to virus infections are primarily characterized by a set of disease symptoms such as dwarfism, increased or reduced tillering numbers; leaves being dark green, yellow, or mosaic; inferior heading; and mostly unfilled grains. Most rice viral diseases can be readily distinguished by these symptoms. Here, major disease symptoms including plant height, tillering number, and abnormal leaf morphology exhibited on rice plants infected by distinct rice viruses are listed in Table 2.

## 4. Transmission Biology

Rice viruses are transmitted by distinct insects or fungi, and most of them cannot be transmitted mechanically or by seeds [69]. RNMV and RSNV are soilborne and is transmitted by the fungus *Polymyxa graminis* [11]. RYMV is transmitted in a semi-persistent manner by various chrysomelid beetles, including *Sesselia pussilla*, *Chaetocnema pulla*, and *Trichispa sericea* [32,70]. It is also mechanically transmissible by the long-horned grasshopper *Conocephalus merumontanus*, as well as by mammals (rats, donkeys and cows) and by birds [71,72]. Other known rice viruses can be transmitted by more than one vector with varying transmission capabilities in rice plants. As to the vector-borne rice viruses, there exist two main horizontal transmission modes: persistently transmitted or semi-persistently transmitted. Persistently transmitted plant viruses are further divided into two groups: propagative and non-propagative viruses, whereas known rice viruses are mostly propagative. To date, how rice viruses replicate or propagate in the body of vector insects have been largely described [73,74,75,76,77]. Basically, to achieve propagation, after virus acquisition from phloem sap of virus-infected rice plants via the stylet, viruses must overcome multiple tissue or membrane barriers in the insects to infect, replicate and finally transmit the virions into healthy plants through saliva secretion, or into the female ovary to be vertically transmitted to offspring [78]. Thereby, for successful virus transmission, each propagative rice virus needs a circulative period in the corresponding vector body. For a semi-persistent transmitted virus, a retain time of viruses in the insects has been evaluated. Here, we summarized the transmission biology of distinct rice viruses in Table 3. The detailed information was mostly collected from two online websites: Descriptions of Plant Viruses (www.dpvweb.net (accessed on 10 September 2022)) and CAB International (www.cabi.org/isc/ (accessed on 10 September 2022)).

## 5. The Complex Interactions between Rice Viruses and Their Hosts

During the last two decades, the devastating damage on rice production by rice viral diseases has driven a sustained quest for rice viral diseases. Two central points involving rice–virus interactions and vector–virus interactions are explored to comprehensively understanding the complex biological processes during virus infection, from which vital insights into disease prevention and control are providing [79,80,81].

**Figure 2:**
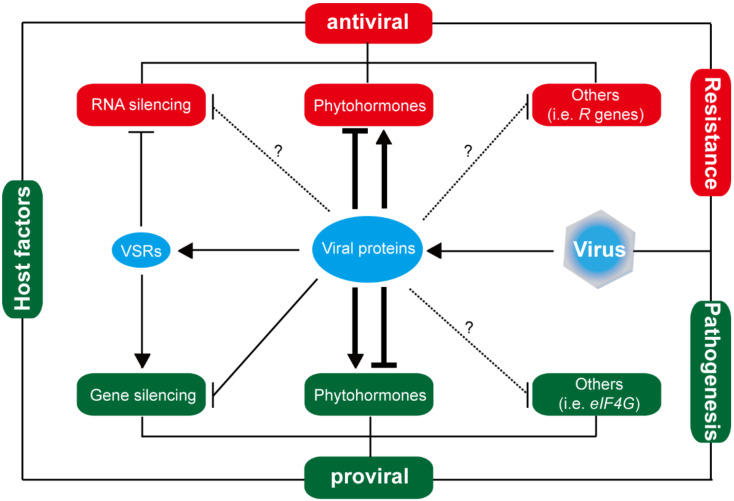
The arms race between rice plants and viruses. Viral proteins execute all parts of virus infection cycle in host cells. They usurp host factors and resources and suppress host defense signals for virus pathogenesis (proviral). Antiviral resistance consists of host factors that target viral proteins or nucleic acids to restrict virus infection (antiviral). The dotted lines and question mark mean there exists uncovered correlations between viral proteins and host factors required for virus pathogenesis or antiviral defense.

To resist virus infection, plants have evolved several defense pathways such as RNA silencing, phytohormone, and plant innate immune system [82,83,84,85]. To establish infection, viruses escape from or suppress antiviral defense by viral silencing suppressors (VSRs) or other viral pathogenic factors [82,85]. These findings indicate an arms race between viruses and rice plants (Figure 2). Herein, a series of host factors associated with RNA silencing, phytohormone, antiviral loci or other signal pathways in rice plants are illustrated (Table 4).

### 5.1. RNA Silencing: The Focus of Rice Defense and Viral Counter-Defense

RNA silencing is one of the most important antiviral pathways for plants to resist virus infection [83,84,85]. In the model plant Arabidopsis, the antiviral RNAi has been well-established through genetic analysis. It relies mainly on the existence of multiple copies of *Dicer-like* (*DCL*), *RNA-dependent RNA polymerase* (*RDR*), *double-stranded RNA binding* (*DRB*), and *Argonaute* (*AGO*) genes [124]. Thereinto, DCLs are involved in the generation of virus-derived small interfering RNAs (vsiRNAs) via the cleavage of viral genome-derived dsRNA structures hierarchically [125]. RDRs are involved in the defense against RNA viruses and production of viral secondary siRNAs [126,127,128]. DRBs might act as viral invasion sensors and contribute to the triggering of various defense responses [129,130]. AGOs can load vsiRNAs to form RNA-induced silencing complexes (RISCs), by which the viral messenger RNAs are targeted with the guiding of vsiRNAs and virus replication is thus limited via a variety of mechanisms such as transcript degradation and translation inhibition [131].

To date, several RNA silencing-associated components have been verified in rice plants by genetic analysis or protein-protein interactions. AGO18, a specific Argonaute protein in monocotyledons, mediates broad-spectrum resistance to virus infections as a molecular lock to binding miR168, thus leading to the alleviated repression of rice AGO1 [86]. They further determined the antiviral role of AGO1 by the finding that knockdown of *AGO1a/1b* expression increases the susceptibility to RSV. In contrast, AGO2 enhances rice susceptibility to fijivirus infection through the DNA methylation-controlling transcription inhibition of *OsHXK1*, by which the ROS-mediated resistance is inactivated [87]. Up-regulation of miR444 diminishes the repressive effects of OsMADS23, OsMADS27a, and OsMADS57 on rice *RDR1* transcription, thus activating the RDR1-dependent antiviral RNAi against RSV infection [88]. Rice plants with down-regulated *RDR6* transcripts are more susceptible to both RSV and RDV, indicating its function in host defense [89,90].

To counter-defend antiviral RNA silencing, rice viruses versatilely impede antiviral RNA silencing signals by encoding VSRs or other functional proteins. For instance, Pns10, a VSR encoded by RDV, can inhibit both local and systemic RNA silencing, possibly by inhibiting the expression of *RDR6* [90]. Pns11, the second VSR of RDV, can effectively suppress local RNA silencing triggered by sense GFP mRNA [132]. NS3, a well-documented VSR encoded by RSV, suppresses RNA silencing by forming dimeric complexes to bind long dsRNA [133]. Both P2, a weak VSR of RSV [91] and RSMV P4 [92] can interact with rice Suppressor of Gene Silencing 3 (SGS3), an essential co-factor function in RDR6-mediated RNAi signal cascade amplification. In addition, RYSV P6 directly interacts with RDR6 for blocking vsiRNA amplification [93]. The present findings indicate that inhibition of vsiRNA generation through sequestering the virus-derived dsRNA or hindering the SGS3/RDR6-mediated vsiRNA amplification is essential for antiviral defense in rice. Besides, some virus-encoded proteins can suppress host defense or mediate the induction of disease symptoms through directly modulating host RNA silencing pathways, particularly miRNA and DNA methylation. It is a universal response that miRNAs are regulated upon virus infection. For instance, RSV NS3 regulates the processing of certain resistance-related miRNAs (i.e., miR168, miR528, miR395, miR398 and miR399) through association with the miRNA biogenesis factor OsDRB1 [94]. RSV infection reduces the protein level of rice SQUAMOSA Promoter Binding Protein-like 9 (SPL9), which serves as the transcription factor to control miR528 transcription, thus the miR528-mediated antiviral defense is subdued [95]. RSV infection also inhibits the accumulation of miR171b, thus causing stunting with reduced chlorophyll content in leaves like viral symptoms [96]. RGSV p3 suppresses RNA-dependent DNA methylation (RdDM) through degrading OsNRPD1a, one of the largest subunits of Pol IV, in a ubiquitination dependent manner, thus facilitating virus infection [97].

Either antiviral RNA silencing and the counter-defense performed by VSRs or other viral proteins indicated that the host RNA silencing pathway is indeed a primary way for rice plants to resist virus infection. While rice genome consists of multiple copies of AGOs, DCLs, RDRs, and other RNAi-associated proteins, thereby more antiviral RNAi components in rice need to be determined in the future.

### 5.2. Plant Hormones: Another Center Target for Various Rice Viruses

Plant hormones exist within plants, contributing to growth and development, but their biosynthesis and signaling can be disrupted or modulated after virus invasion [134,135]. During the last decade, plant hormones seem to be rising stars for the studies in rice–virus interaction.

Thus far, the well-known plant hormones, including gibberellic acid (GA), salicylic acid (SA) [136], jasmonic acid (JA) [137], auxin (Aux) [138], ethylene (Et), abscisic acid (ABA) [98], brassinosteroid (BR), and strigolactone (SL), have been demonstrated to be closely related to virus pathogenesis or host defense. Several phytohormone-associated components were found to be linked to symptom development of various viruses. For instance, the RDV-caused dwarf symptom in rice is because RDV-encoded P2 can reduce biosynthesis of GA through the interaction with rice ent-kaurene oxidases or ent-kaurene oxidase-like proteins [139]. Further research indicated that P2 also hijacks auxin signaling by directly targeting the rice OsIAA10 to induce symptom development such as dwarfing, more tillering number, shorter crown roots, and lower fertility rates [36]. Moreover, RGSV-induced symptoms, including stuntedness and excessive tillering, are partly because of the inhibition of SL accumulation or signaling by P3-mediated post-transcriptional gene silencing of SL-associated genes [97].

Viruses or viral proteins also directly or indirectly target distinct phytohormone-associated components to promote virus infection [85]. RDV Pns11 promotes host SAMS1 activity and ethylene production for the benefit of virus infection [99]. ARF17, a positive transcription factor involved in auxin signaling, is targeted by distinct viral proteins, including P8 encoded by SRBSDV and RBSDV, P2 encoded by RSV, and P4 encoded by RSMV, thereby leading to the impairment of OsARF17-mediated antiviral defense [100]. RBSDV P5-1 can regulate activity of SCF E3 ubiquitin ligases through interaction with OsCSN5A, and inhibit JA signaling pathway to benefit its infection in rice [101]. To facilitate virus infection, JA pathway is targeted by RRSV, by which miR319 was highly induced and its targeting gene *TCP21* was down-regulated for suppression of JA biosynthesis and signaling [102]. Recently, JA signaling positively regulated antiviral RNA silencing was uncovered, suggesting a link between phytohormone-mediated antiviral defense and RNA silencing-mediated antiviral defense. JA signaling is activated upon RSV infection, followed by degradation of JAZ6 and induction of transcription factor JAMYB, by which the promoter activity of AGO18 was directly regulated and AGO18 expression was induced [103]. Sometimes, different plant hormone signals cross talk after virus invasion [104]. For instance, both RBSDV and RSV can induce the expression of the kinase OcGSK2, a key suppressor involving BR signaling. The induction of OsGSK2 promotes the phosphorylation and degradation of transcription factor OsMYC2, which leads to the inactivation of JA-responsive genes and enhances rice susceptibility to RSV infection [105]. However, upon RBSDV infection, the induction of OsGSK2 positively regulates the JA antiviral response to RBSDV by promoting the phosphorylation and degradation of OsJAZ4, a repressor of the JA signaling [105]. During the early stage of SRBSDV infection, SRBSDV P6 activates ethylene signaling via interacting with OsRTH2 in the cytoplasm, thus leading to enhanced SRBSDV proliferation [106]. These findings imply positive roles of JA and auxin for antiviral defense, but ethylene contributes to virus proliferation.

### 5.3. Antiviral Loci from Rice Genome

By forward genetic approaches, a sulfotransferase, STV11, with function in catalyzing the conversion of SA to sulphonated SA, is identified as an available anti-RSV locus [107]. To screen RBSDV resistance genes, a diverse global collection comprising 1,953 rice accessions was evaluated under natural conditions across 3 years [108]. Further haplotype analyses suggested that a candidate gene *LOC_Os06g03150* is mainly associated with the differentiation of resistance within the *Xian* subgroup and *LOC_Os06g31190* mainly explained the difference in the resistance between *Xian* and *Geng*. Using genome-wide association study (GWAS), linkage disequilibrium (LD) decay analyses, RNA-sequencing, and genome editing, a highly RBSDV-resistant variety and its first functional gene, Hap1, was identified [140]. Besides, multiple recessive resistance loci to RYMV have been mapped [141,142]. For example, mutations in the eIF(iso)4G translation initiation factor confer high resistance of rice to RYMV [109]. Furthermore, a second major resistance gene to RYMV, RYMV2, was screened from a large scale African cultivated rice species, *O. glaberrima* [112]. Further study indicated that RYMV2-mediated resistance to RYMV is associated with a rice homolog of the *CPR5* gene, a regulator of active defense mechanisms [113]. Later, a new resistance gene to RYMV from *O. glaberrima* was identified through fine mapping method by the same group [114].

### 5.4. Other Host Components Involved in Rice-Virus Interactions

Although the primary plant defense is based in this case mainly on RNA silencing and plant hormones, other cellular pathways are also involved in the rice–virus interactions. Recently, two proteins associated with plant innate immunity, OsRLP1 and OsSOBIR1, are proved to positively regulate rice immunity against RBSDV infection, suggesting pathogen-triggered immunity (PTI)-based innate responses could contribute to antiviral plant defense [115]. Our recent work proved that overexpression of *OsHAK5* potassium transporter improves virus resistance against RGSV infection [116]. Besides, CBL-interacting protein kinase 25 (OsCIPK25), a member of the plant-specific CBL-CIPK Ca^2+^ signaling network, has been proven to interact with RGSV p5 [117]. PsbP, a 23-kDa oxygen-evolving complex protein, can be interacted by RSV-encoded disease-specific protein (SP), thus leading to enhanced virus symptoms [118]. To overcome remorin-mediated inhibition of virus movement in host plants, RSV decreases the remorin protein level by interfering with its S-acylation [119]. These findings suggest that host components involved in plant innate immunity, plant nutrient, plant photosystem, plant-specific membrane-associated proteins and other unknown proteins might be associated with rice–virus interactions.

RSV can be mechanically transmitted to *Nicotiana benthamiana*, thereby a research model of RSV in *N. benthamiana* has been developed and applied. To date, several host factors of *N. benthamiana* with function in regulating RSV infection have been uncovered. Viral proteins can manipulate organelle function to benefit virus infection. For instance, RSV p2 may recruit or manipulate nucleolar functions through an interaction with fibrillarin to promote virus systemic movement [120]. NSvc4 targets host chloroplasts to suppress chloroplast-mediated defense [121]. Besides, silencing of *NbeIF4A* activates autophagy and inhibits RSV infection by facilitating autophagic degradation of p3. Further study indicated that overexpression of vsiRNA-4A, a viral siRNA derived from RSV RNA4, targets *eIF4A* mRNA for cleavage, thus leading to induced autophagy [122]. Recently, RSV activates the bZIP17/28 branch of unfolded protein response signaling pathway to promote viral infection [123]. These findings have promoted the understanding of the infection mechanism of RSV.

### 5.5. Complex Interactions between Rice Viruses and Their Vectors

Unlike the huge adverse on rice growth and development by rice viruses, persistent-propagative infection by rice viruses causes a limited pathogenicity in their vectors. For example, RDV [143], RGDV [144], RSV [145], and SRBSDV [146] reduce the survival rate, longevity and fecundity of their offspring or modify the performance and behavior of their adult vectors. It seems that there exists a balance between the fitness cost of the viral infection of insects and the persistent transmission of the virus by the insect. The virus thresholds in insect vectors have been proved as a key factor for further virus transmission. For example, transmission frequency was correlated with SRBSDV or RBSDV content in the salivary gland of vector [147]. In contrast, treatment by ribavirin, with function in specifically inhibiting the expression of sugar transporter 6, a key vector component for RSV transmission, leads to less RSV accumulation in *L. striatellus* tissues and thereby lower transmission efficiency [148]. Then, how to keep the virus at a optimal level in the body of vector insects? During the last decades, this question has been largely addressed through investigating replication cycle of rice viruses in their vector insects and the complex interactions between rice viruses and their vectors [81,149,150].

Persistent transmission by insects firstly needs a successful initiation of viral replication cycle comprising of virus entry, multiplication, and spread in their insect vector cells [149]. Many virus-encoding proteins involving in the replication cycle have been determined. For example, RDV P2, protruding from the surface of the outer shell of virions, is essential for virions entry into insect vector cells in a receptor-mediated, clathrin-dependent endocytosis manner [151,152,153,154]. Nonstructural proteins such as RBSDV P9-1 [155,156], RDV Pns12 [157], RGDV Pns9 [158], RRSV Pns10 [77,159], and RYSV P6 [160] contribute to the assembly of viroplasm for viral genome replication and progeny virion assembly. After entry and replication, viruses counter two main physical barriers in the insect vectors for spread: midgut barriers and salivary gland barriers. Nonstructural proteins such as SRBSDV and RBSDV P7-1 [161,162,163], RDV Pns10 [164], and RGDV Pns11 [165] have the capacity to form homodimers to assemble the proposed helical symmetry structures of tubules, by which virions can rapidly disseminate from the intestine barriers of the insect vector. This mechanism is known as ‘actin-based tubule motility’. The understanding of how viruses overcome the plasmalemma barrier of the salivary gland cavities in insect vectors is limited. In the case of RGDV, virus-induced filaments to perform an exocytosis-like process that enables viral passage through the apical plasmalemma into salivary cavities [166].

Apart from pass through the physical barriers in the insects, rice viruses also manipulate vector immune systems for efficient infection of insect cells and persistent transmission by insect vectors. Autophagy pathway is always modulated by rice viruses and often positively regulates persistent viral propagation in insect vectors [167]. For example, RGDV replication in cultured leafhopper vector cells and intact insects can trigger the autophagic process such as obvious virus-containing double-membrane autophagosomes, conversion of ATG8-I to ATG8-II and increased level of autophagic flux, by which the viral spread in the leafhopper intestine is facilitated [168]. The positive roles of autophagy in persistent transmission of rice viruses by insect vectors are further supported by a series of research projects recently. RGDV P2 directly recruits a GAPDH-ATG4B complex to induce the formation of initial autophagosomes, which were further modified to evade fusion with lysosomes for degradation, and persistently exploited by viruses to promote efficient propagation [169]. They also found that RGDV Pns11 directly recruits ATG5-ATG12 conjugation to induce the formation of autophagosomes, thereby facilitating viral spread within the insect bodies [170]. SRBSDV P7-1 interacts with the mitophagy receptor BNIP3 (BCL2 interacting protein 3) and induces BNIP3-mediated mitophagy by bridging autophagosomes and mitochondria for maintaining persistent viral infection in insect vectors [171]. But sometimes, autophagy negatively regulates virus infection in insects. A recently study exhibits that RBSDV hijacks PtdIns(3,5)P2 via its viral capsid protein to evade autophagic degradation and promote its survival in insect cells [172]. These findings suggest autophagy may serve as a double-edged sword during viral infection. Rice viruses such as RGDV and RRSV also induce apoptosis in insect vector cells [173,174], suggesting a link between virus-induced apoptosis and virus transmission insect ve1ctors to the host plant. RSV CP also competitively bound G protein Pathway Suppressor 2, an inhibitor of the c-Jun N-terminal kinase (JNK) signaling, thus releasing the JNK signaling to promote RSV replication in the vector [175]. Although cytopathologic alterations such as autophagy or apoptosis are triggered to regulate virus persistent transmission, their potential damage on the relevant tissues and organs may alter the fitness of the insect vectors.

Antiviral RNAi pathway, a primary antiviral response in plants, can also be triggered by the propagation of plant viruses in insect vectors, evidenced by the characterization of vsiRNAs in virus-infected insect vector [176,177]. Antiviral RNAi in insect vectors can control the initial infection of the insect midgut epithelium, which eventually affects the vector competence for the virus. A recent report also indicates a novel control strategy of viral replication in insect vectors whereby interaction between endogenous microRNAs and vsiRNAs [178]. They found that the transcription of miR-263a was suppressed by RSV-derived vsR-3397, thus leading to a compromised RSV replication and was beneficial for maintaining a tolerable accumulation level of RSV in insect vectors. Notably, RNA modification also correlates with viral persistence in insect vectors. For example, N^6^ methylation of adenosine (m^6^A) regulates RBSDV replication in its vector via modifying viral and insect RNA, and it is used by the virus to maintain a specific titer threshold required for viral transmission [179]. This regulation mechanism either on protein level or RNA level reflects an ingenious adaptation strategies of viruses to their insect vectors.

Such transovirial viruses that RDV [180,181], RGDV [182,183], and RSV [184] vertically transmit to vector offspring via exploiting a symbiotic bacterium, directly hitchhiking with insect sperm without disturbing sperm functioning, using vitellogenin-mediated transovarial transportation system, or entering the oocyte of insect vector via virus-induced tubules [78]. These findings verify that viral vertical transmission of a rice virus by insect vectors in nature exists two modes: maternal transmission and paternal transmission.

Emerging evidence has proven that rice viruses can manipulate their vectors either directly or by inducing changes in host plants to promote the spread of viral pathogens. For example, during the late infection of SRBSDV in rice plants, SRBSDV P6 enters the nucleus and interacts with OsEIL2 to suppress ethylene signaling by blocking the dimerization of OsEIL2, thus promoting virus spread via attracting the insect vector [106]. It implies more complex interactions among insect vectors, viruses, and host plants contribute the transmission of viruses by balancing all organizational levels, from molecules to populations. Once one or more of the interactions undergo changes through evolution or are halted by environmental interventions, virus transmission may be failed. That’s a quite possible reason for explaining why field epidemics of rice viral diseases present three typical characteristics: outbreak, intermittence, and migration. Thereby, exploration of rice-virus-vector should be focused for seeking solutions to control rice viral diseases.

## 6. Disease Prevention and Control Strategies for Rice Viral Diseases

With the long-term efforts of studying on rice viral diseases, many strategies for disease prevention and control have been developed as a system. By collecting the present knowledge, four main points are summarized as follows.

### 6.1. Generation of Resistant Rice Varieties

Generation of resistant rice varieties through different breeding strategies is always a more safe and effective way to prevent and control rice viral diseases. While owing to the limitation of antiviral gene resources, antiviral breeding for disease resistance processes slowly. But many other strategies for resistance to viral diseases have been employed, including encapsulation of viral genome, silencing of viral genes by antisense RNA or by small RNA molecules, expression of ribozyme, or modification of host factors. For example, as early as in 1999, transformed lines with expressing the RNA-dependent RNA polymerase of RYMV were found to be resistant to RYMV strains from different African locations, sometimes there were complete suppression of virus multiplication [185]. In addition, expressing hairpin RNA targeting viral genes can limit plant viruses in rice to a great degree [186,187,188,189]. Generation of transgenic rice plants expressing artificial miRNA directly targeting Virus Genes also possess high-effective disease resistance. For example, transgenic rice plants expressing artificial miRNA targeting RSV *MP* are highly resistant to the virus [190]. Recently, marker-free rice plants with stable and high resistance to RBSDV through RNA interference were also developed [191]. These marker-free transgenic lines driven by either maize ubiquitin-1 promoter or rice rbcS green tissue expression promoter in elite rice background should have great potential in breeding of resistant varieties to both RBSDV and SRBSDV. Another strategy that to modify host factors with function in helping virus propagation has already successfully established. For example, genome editing of rice eIF4G loci through CRSPR/Cas9 method conferred partial resistance to both RTSV and RBSDV [110,111]. By applying CRISPR/Cas13a immune system, virus resistance in both dicot and monocot plants were obtained [192]. Additionally, the integration of current miRNA knowledge with conventional and modern crop improvement strategies is a method to generate resistant rice varieties [193,194].

Although many strategies are exercisable and effective to generate resistant rice varieties, rare varieties are generated yet. Even worse, some identified resistance loci have potential ability to be overcome by the evolution of virus genome. For example, insertion of an E309K mutation in *eIF(iso)4G1*, the well-established resistance locus in rice, strongly diminished the interaction with avirulent VPg [195]. These situations urge us that more potential resistance gene resources need to be determined and applied to breeding work in the future. There is still a way to go to open a new paradigm in rice breeding by closely combining resistance cultivars to various rice viruses.

### 6.2. Adapt Measures to Avoid Virus Infection at Seedling Stage

Rice plants are more susceptible to viruses at the seedling stage. For example, rice is susceptible to SRBSDV infection during all growth periods, but the symptoms depend on the growth stage at the time of infection, and the earlier the infection occurs, the more severe the symptoms are [50,196]. Thus, knowing how to protect rice seedlings in seedling nurseries will greatly alleviate disease occurrence and prevalence. Solutions established in different rice-growing areas are illustrated as follows: (1) in the tropical rice-growing region, use of planting dates that avoid high vector populations will largely reduce diseased seedlings; (2) covering rice seedling nurseries with insect-proof nets to segregate rice seedlings with viruliferous insects [197]; (3) cultivating rice seedlings in greenhouse and then transplanting in the fields. These practices are the most economical and effective preventive measure for rice viral diseases.

### 6.3. Use Pesticides Rationally to Reduce Transmission Agents

Pesticides should first be applied in rice fields to control overwintering or the first-generation of vector, and in rice seedling nurseries to reduce the population of insect vectors, thereby the primary source of infection largely decreased [51]. For example, once overwintering or the first-generation of *R. dorsalis* were controlled by applying pesticides, the RSMV transmission was largely suppressed. For RYMV, a soil-borne rice virus, seed pelleting, or coating seedling is recommended to control the rice infection in seedling nurseries.

### 6.4. Development of Commercial Antiviral Agents

It is of great importance to develop safe and effective chemical agents for controlling plant viral diseases. To date, several immune stimulants such as Dufulin, Amino oligosaccharide, and moroxydine hydrochloride have been developed and applied into viral disease control [198]. However, the species of commercial antiviral agents are too scanty. A recent paper proposed viral coat proteins as biochemical targets for antiviral compounds [199], which hopefully provides new insights and references for further development of new safe and effective antiviral pesticides.

## 7. Conclusions and Future Perspectives

This review primarily summarizes the occurrence and damage of rice viral diseases, disease symptoms, transmission biology, arms race between rice viruses and rice plants as well as insect vectors, and the strategies in viral disease management. This knowledge tells us about a major challenge during the coming decade that how to balance rice production and consumption.

Repeated outbreaks of known and newly emerged rice viruses remind us that rice viruses are tricky. We know little about their law of existence: how they come like SRBSDV emerging since 2001 in China, and how they leave or stash in the vast nature such as RBSV. Most important, under natural conditions, life system of crops, biotic factors, abiotic factors, and human activities should be balanced in the agroecosystem. Once such balance is stricken by external or internal elements such as environment, plant nutrients, transmission ability of vectors, weak link will be vulnerable to cause the outbreak and epidemic disease [200,201,202]. These existing questions have prompted us to accelerate our capacity and progress in researching rice virus diseases. As the propose raised by Christopher Surridge 20 years ago, feeding the world in the 21st century urgently requires a second green revolution, involving the most audacious feat of genetic engineering yet at the top of the waves [203]. These have been hailed as the magic bullet that will help to fulfil the demand for increasing consumption and finally solve the world’s food crisis substantially.

## Figures and Tables

**Table 1:** Known rice viruses in rice-growing areas worldwide.

Geographic Range	Virus Name	Genus and Family	Location of First Report	Year of First Report	Reference
Asia	rice dwarf virus	*Phytoreovirus*, *Sedoreoviridae*	Japan	1883	[10,11]
	rice stripe virus	*Tenuivirus*, *Phenuiviridae*	Japan	1897	[12]
	rice black-streaked dwarf virus	*Fijivirus*, *Spinareoviridae*	Japan	1952	[13]
	rice grassy stunt virus	*Tenuivirus*, *Phenuiviridae*	Philippines	1963	[14]
	rice tungro bacilliform virus	*Tungrovirus*, *Caulimoviridae*	Philippines	1963	[15,16]
	rice tungro spherical virus	*Waikavirus*, *Secoviridae*			
	rice yellow stunt virus	*Alphanucleo-rhabdovirus*, *Rhabdoviridae*	Guangdong and Taiwan, China	1965	[17,18,19]
	rice bunchy stunt virus	*Phytoreovirus*, *Sedoreoviridae*	China	1976	[20]
	rice ragged stunt virus	*Oryzavirus*, *Spinareoviridae*	Philippines	1977	[21]
	rice gall dwarf virus	*Phytoreovirus*, *Sedoreoviridae*	Thailand	1979	[22]
	rice necrosis mosaic virus	*Bymovirus*, *Potyviridae*	Japan	1977	[23,24]
	southern rice black-streaked dwarf virus	*Fijivirus*, *Spinareoviridae*	China	2008	[25,26]
	rice stripe mosaic virus	*Cytorhabdovirus*, *Rhabdoviridae*	China	2017	[27,28]
	rice virus A (tentative name)	Unassigned, *Tombusviridae*	Korea	2017	[29]
	rice curl dwarf-associated virus (tentative name)	Unassigned	China	2021	[30]
	rice dwarf-associated bunya-like virus (tentative name)	Unassigned, closed to *Discoviridae*	China	2022	[31]
Africa	rice yellow mottle virus	*Sobemovirus*, *Solemoviridae*	Kenya	1966	[32]
Africa and America	rice stripe necrosis virus	*Benyvirus*, *Benyviridae*	Côte d’Ivoire	1983	[33,34]
America	rice hoja blanca virus	*Tenuivirus*, *Phenuiviridae*	Colombia	1935	[10,11]

Note: The genus and family of each virus are portrayed according to the 10th ICTV report.

**Table 2:** Disease symptoms induced by different rice viruses.

Virus Name	Symptoms		
	The Whole Plant	Tillering Number	Leaf
RDV	Dwarfing	Increased	Short leaves that are dark green in color with fine chlorotic specks
RSV	Slightly dwarf	Normal	Faded green stripe or mottling and necrotic streaks
RBSDV	Dwarfing	Increased	Darkening of leaves, twisting of leaf tips, splitting of the leaf margin, and waxy white-to-black galls along the veins on the underside of leaf blades
RGSV	Stunted	Increased	Short, erect, and narrow leaves that are pale green or pale yellow in color
RTBV	Stunted	Decreased	Yellow or orange discoloration
RTSV			
RYSV	Mild stunted	Reduced	Yellow, wilting or dead leaves
RBSV	Stunted, bird nest-like	Increased	Dark green and narrow leaves
RRSV	Stunted	Increased	Dark green, ragged leaves with vein swelling or galls on the underside of leaf blades
RGDV	Dwarfing	Decreased	Short darker green leaves, and small galls along the leaf veins on the undersurface of leaves
RNMV	Moderately stunted	Reduced	Yellow flecks and streaks on lower leaves
SRBSDV	Dwarfing	Increased	Dark green leaf and small enations on leaf back
RSMV	Slightly dwarfing	Increased	Twisted leaves exhibiting striped mosaicism
RVA (tentative name)	Not described	Not described	Not described
RCDaV (tentative name)	Dwarfing	Not described	Leaf curling
RDaBV (tentative name)	Dwarfing	Decreased	Not described
RYMV	Stunted	Decreased	Yellow mottle or orange coloration
RSNV	Dwarfing	Reduced	Chlorotic or yellow stripes on leaves, which laterbecome necrotic
RHBV	Dwarfing	Decreased	Chlorotic or yellow striping and mottling of leaves, and premature wilting or dead

**Table 3:** Transmission biology of distinct rice viruses.

Virus Name	Modes of Transmission	Circulative Period/Retain Time (Day)	Vector Species
RDV	Persistent-propagative, transovarial	12–25	***Nephotettix cincticeps***, *Recilia dorsalis*, *Nephotettix virescens*, *Nephotettix nigropictus*
RSV	Persistent-propagative, transovarial	5–21	***Laodelphax striatellus***
RBSDV	Persistent-propagative	4–35	***Laodelphax striatellus***, *Nilaparvata lugens*, *Unkanodes albifascia*, *Unkanodes sapporonus*
RGSV	Persistent-propagative	3–28 (8)	***Nilaparvata lugens***, *Nilaparvata bakeri*, *Nilaparvata muiri*
RTBV	Semi-persistent	4–5	*Nephotettix cincticeps*, *Nephotettix malayanus*, *Nephotettix nigropictus*, ***Nephotettix virescens***, *Recilia dorsalis*
RTSV	Semi-persistent	2–4	*Nephotettix cincticeps*, *Nephotettix malayanus*, *Nephotettix nigropictus*, ***Nephotettix virescens***, *Recilia dorsalis*
RYSV	Persistent-propagative	Undetermined	*Nephotettix cincticeps*, ***Nephotettix nigropictus***, *Nephotettix virescens*
RBSV	Persistent-propagative	11	***Nephotettix cincticeps***, *Nephotettix virescens*
RRSV	Persistent-propagative	2–33 (9)	***Nilaparvata lugens***
RGDV	Persistent-propagative, transovarial	8–18	*Nephotettix cincticeps*, *Nephotettix malayanus*, *Nephotettix nigropictus*, *Nephotettix virescens*, ***Recilia dorsalis***
RNMV	/	/	***Polymyxa graminis***
SRBSDV	Persistent-propagative	6–14	***Sogatella furcifera***
RSMV	Persistent-propagative	8–16	***Recilia dorsalis***, *Nephotettix virescens*
RVA (tentative name)	Unknown	Unknown	Unknown
RCDaV (tentative name)	Unknown	Unknown	Unknown
RDaBV (tentative name)	Unknown	Unknown	Unknown
RYMV	Semi-persitent, non-circulative	1–8 (2–3)	***Sesselia pussilla***, *Chaetocnema pulla*, *Trichispa sericea*
RSNV	/	/	***Polymyxa graminis***
RHBV	Persistent-propagative, transovarial	4–31	***Sogatodes oryzicola***, *Sogatodes cubanus*, *Tagosodes orizicolus*

Note: The usual length of the circulative period of certain viruses is indicated in parentheses. The major transmission vector for each virus is highlighted in bold type.

**Table 4:** Host factors with antiviral activity against rice viruses.

Host Factor	Cellular Function	Virus	Viral Factor	Technique	Reference
AGO1	Antiviral RNA silencing	RSV, RDV	Undetermined	Genetic analysis	[86]
AGO2	Epigenetic regulation	RBSDV, SRBSDV	Undetermined	Genetic analysis	[87]
AGO18	Antiviral RNA silencing	RSV, RDV	Undetermined	Genetic analysis	[86]
RDR1	Antiviral RNA silencing	RSV	Undetermined	Genetic analysis	[88]
RDR6	Antiviral RNA silencing	RSV, RDV, RYSV	Pns10, P6	Genetic analysis, protein-protein interaction	[89,90,91]
SGS3	Antiviral RNA silencing	RYSV, RSV, RSMV	P2, P4	Transient expression, protein-protein interaction	[92,93]
DRB1	miRNA process	RSV	NS3	Transgenic method	[94]
SPL9	miRNA transcription	RSV	Undetermined	Transgenic method	[95]
miR171b-SCLs	Gene silencing	RSV	Undetermined	Transgenic method	[96]
HXK1	ROS	RBSDV	Undetermined	Transgenic method	[87]
Pol IV	RdDM	RGSV	P3	Transgenic method, yeast two-hybrid	[97]
KO2, KOL4, KOL5	GA	RDV	P2	Yeast two-hybrid	[98]
IAA10	Aux	RDV	P2	Transgenic method	[36]
SAMS1	Et	RDV	Pns11	Transgenic method	[99]
ARF17	Aux	RBSDV, SRBSDV, RSV, RSMV	P8, P2, P4	Transgenic method, protein-protein interaction	[100]
CSN5A	JA	RBSDV	P5-1	Protein-protein interaction	[101]
miR319-TCP21	Gene silencing, JA	RRSV	Undetermined	Transgenic method	[102]
JAMYB	JA	RSV	Undetermined	Transgenic method	[103]
GSK2	BR-JA	RSV, RBSDV	Undetermined	Transgenic method	[104,105]
RTH2 and EIL2	Et	SRBSDV	P6	Transgenic method, protein-protein interaction	[106]
STV11	SA	RSV	Undetermined	Genetic analysis	[107]
Hap1	Amino acid metabolism	RBSDV, SRBSDV	Undetermined	GWAS, LD decay analyses, RNA-sequencing, and genome editing	[108]
eIF4G	Translation initiation	RYMV, RTSV, RBSDV	RYMV VPg	Genetic analysis, transgenic method	[109,110,111]
CPR5-1	Defense mechanism regulator	RYMV	Undetermined	Fine mapping	[112,113]
RYMV3	Unknown	RYMV	Undetermined	Fine mapping	[114]
RLP1 and SOBIR1	PTI response	RBSDV	Undetermined	High-throughput-sequencing, genetic analysis	[115]
HAK5	Potassium transport	RGSV	P3	Transgenic method	[116]
CIPK25	Ca^2+^ signaling	RGSV	P5	Protein-protein interaction	[117]
PsbP	Photosystem	RSV	SP	Protein-protein interaction	[118]
REM1.4, NbREM1	Plant-specific membrane-associated protein	RSV	NSvc4	Transgenic method, Protein-protein interaction	[119]
Nbfibrillarin	Nucleolar functions	RSV	P2	VIGS, protein-protein interaction	[120]
NbGAPDH-A and NbPsbQ1	Chloroplast functions	RSV	NSvc4	VIGS, protein-protein interaction	[121]
NbeIF4A and NbATG5	Autophagy	RSV	vsiRNA-4A	VIGS, protein-protein interaction	[122]
NbbZIP17/28	Unfolded protein response signaling	RSV	Undetermined	VIGS	[123]

## Data Availability

Not applicable.
